# Lung Cancer: Targeted Therapy in 2025

**DOI:** 10.3390/curroncol32030146

**Published:** 2025-03-02

**Authors:** Nicole Bouchard, Nathalie Daaboul

**Affiliations:** 1 Centre Hospitalier Universitaire de Sherbrooke, Université de Sherbrooke, Sherbrooke, QC J1H 5N4, Canada; nicole.bouchard@usherbrooke.ca; 2 Centre Intégré de Cancérologie de la Montérégie, Université de Sherbrooke, Longueuil, QC J4V 2H1, Canada

**Keywords:** lung cancer, molecular testing, targeted therapy

## Abstract

Lung cancer treatment has changed in the last twenty years since the discovery of EGFR mutations. In this article, we will review the current state of the art for non-small cell lung cancer (NSCLC) actionable genomic alterations (AGA). AGAs are mostly found in lung adenocarcinomas, a subtype of non-small cell lung cancers. We will focus on the current treatment for EGFR mutations, ALK fusions, ROS1 fusions, BRAF V600E mutations, MET exon 14-skipping mutations, RET fusions, KRAS G12C mutations, ERBB2 mutations (also called HER2 mutations), and NTRK fusions. We will also touch on the key toxicities associated with these medications. Treatments are mostly available for the metastatic stage, but we will also discuss adjuvant therapy for EGFR mutations and ALK fusions, as well as stage III post-chemoradiotherapy treatment for EGFR lung cancer.

## 1. Introduction

Lung cancer treatment has changed in the last twenty years since the discovery of EGFR mutations. In this article, we will review the current state of the art for non-small lung cancer actionable genomic alterations (AGAs). AGAs are mostly found in lung adenocarcinomas, a subtype of non-small cell lung cancers. The prevalence of different AGAs varies (Table 1), with the most common being EGFR. We will focus on the current treatment for EGFR mutations, ALK fusions, ROS1 fusions, BRAF V600E mutations, MET exon 14-skipping mutations, RET fusions, KRAS G12C mutations, ERBB2 mutations (also called HER2 mutations), and NTRK fusions. We will also touch on the key toxicities associated with these medications. Treatments are mostly available for the metastatic stage, but we will also discuss adjuvant therapy for EGFR mutations and ALK fusions, as well as stage III post-chemoradiotherapy treatment for EGFR lung cancer.

## 2. Rationale of Molecular Testing

Molecular testing is essential to better assess the lung cancer signature and identify genomic alterations [1]. It can take many forms, including immunohistochemistry (IHC), fluorescence in situ hybridization (FISH), point mutations detected by PCR, and, most recently, next-generation sequencing (NGS). Experts recommend performing a broad panel to identify alterations in current and soon-to-be available therapies. This also highlights the importance of aiming for precision medicine in lung cancer and finding appropriate targeted therapies. Testing can be conducted on tissue or a liquid biopsy. PD-L1 is also crucial to obtain (can only be conducted on tissue specimens) and is complementary to molecular testing.

Testing should be offered for all non-small cell lung adenocarcinomas and in some squamous cell carcinoma or rarer histological subtypes. This is more relevant for patients with no/little smoking history, where the likelihood of finding an AGA is higher. Statistics show that lung cancer survival has increased with clinical research as new therapies become available, with the most benefit seen with targeted therapy and immunotherapy [1]. In this article, we will mainly review targeted therapy for AGAs.

## 3. EGFR Mutations: Common Mutations (Exon 19 Deletions and L858R)

EGFR mutations are usually detected in lung adenocarcinoma. The common phenotype is in never smokers, females, and Asian ethnicity.

### 3.1. Metastatic Disease

In 2004, an article on mutations in the EGFR underlying the responsiveness of non-small cell lung cancer to gefitinib was published in the New England Journal of Medicine [2,3]. It was the first AGA described in lung cancer and has opened the door to targeted therapy.

Common EGFR mutations include exon 19 deletions and exon 21 L858R. In 2009, the IPASS trial compared gefitinib to chemotherapy (platinum doublet) [4]. A significant benefit was demonstrated in the subgroup of EGFR patients (HR 0.48). Since then, research has been thriving to try to find potent EGFR inhibitors. Many treatments for EGFR metastatic cancer are now available, including erlotinib, afatinib, dacomitinib, and finally, osimertinib (Table 2). Second-generation inhibitors have more adverse effects than first-generation and are less commonly used.

Osimertinib, an oral third-generation EGFR irreversible tyrosine kinase inhibitor (TKI), has been proven to be more effective than gefitinib or erlotinib, which are first-generation TKIs. In the FLAURA trial, osimertinib had a median progression-free survival (PFS) of 18.9 months versus 10.2 months (HR 0.46) for standard TKI [5]. In 2020, the FLAURA trial showed that the median overall survival (OS) was 38.6 months in the osimertinib group compared to 31.8 months (HR 0.80) in the comparator group (gefitinib/erlotinib) [6]. At 3 years, 28% in the osimertinib and 9% in the comparator group continued to receive trial treatment. Adverse events of grade 3 or higher were lower with osimertinib versus the comparator (42% versus 47%). This trial established a new standard of care for metastatic EGFR lung cancer. PFS was also better for patients with brain metastases, since osimertinib crosses the blood–brain barrier.

EGFR TKIs are generally well-tolerated, and the side effects are manageable. They also usually offer a better quality of life compared to chemotherapy. The main toxicities that occur at different rates depending on the TKI prescribed are gastrointestinal (stomatitis, diarrhea), cutaneous (acneiform rash, paronychia), and hepatotoxicity. For osimertinib, electrocardiograms should be regularly monitored for QT interval prolongation.

Unfortunately, some patients will not respond to the drug or will only derive a short-term benefit. There is a need for more effective treatments, especially for patients with brain metastases, L858R mutations, concomitant TP53 mutations, or positive liquid biopsy (also called ctDNA). Two phase 3 trials, FLAURA2 and MARIPOSA, have, respectively, studied the benefit of either adding chemotherapy or amivantamab, a bispecific antibody targeting EGFR and MET. A meta-analysis analyzed the different first-line options and concluded that combination regimens based on third-generation TKIs could be the new preferable first-line standard of care for EGFR-mutated advanced NSCLC [7].

The FLAURA2 trial compared osimertinib with or without chemotherapy (pemetrexed plus either cisplatin or carboplatin) [8]. It showed significant improvement in PFS with the combination of drugs. Median PFS was 25.5 months in the arm with osimertinib with chemotherapy versus 16.7 months (HR 0.62) in the osimertinib alone arm. The overall survival data are still not mature. In the subgroup analysis, some patients seem to be benefiting more from treatment intensification. Among patients with brain metastases at baseline, the median PFS was 24.9 months in the Osimertinib–chemotherapy group and 13.8 months in the osimertinib group. Another subgroup is patients with three or more anatomic sites of metastases (PFS 24.9 versus 16.4 months, HR 0.57) [9]. Adverse events were more frequent in the combination group, due to known chemotherapy adverse events, including hematological toxicities. Experts note that the addition of chemotherapy to osimertinib may be helpful for younger patients with high-burden disease and/or brain metastases.

The MARIPOSA trial compared amivantamab and lazertinib versus osimertinib [10]. Lazertinib is an oral third-generation EGFR irreversible TKI. Amivantamab is a bispecific antibody against EGFR and MET administered by an intravenous infusion. The median PFS was longer in the amivantamab–lazertinib group than in the osimertinib group (23.7 vs. 16.6 months, HR 0.70). Not surprisingly, osimertinib and lazertinib showed similar efficacy. Among patients with known high-risk disease (brain metastases, liver metastases, TP53 co-mutation, and positive ctDNA), amivantamab–lazertinib also improved median PFS [11]. The overall survival data are still not mature. However, a press release announced that amivantamab plus lazertinib shows statistically significant and clinically meaningful improvement in overall survival versus osimertinib [12]. The improvement in median OS is expected to exceed one year. Adverse events were more frequent with the combination of drugs, due to EGFR and MET-related toxic effects. The anticipated toxicities with amivantamab are infusion-related reactions (most frequently on the first cycle), cutaneous (acneiform rash), gastrointestinal (stomatitis), and hypoalbuminemia with peripheral edema. With the combination, venous thromboembolic events were also increased. Prophylactic administration of oral anticoagulant medications during the first 4 months of the regimen decreased the risk of thrombosis and is now recommended.

Eventually, most if not, all patients on EGFR TKIs will progress. The most common resistance mechanisms described after osimertinib are MET amplification, HER2 amplification, and on-target or off-target EGFR mutations. A minority of patients will also experience histological transformation in small-cell lung cancer or squamous cell carcinoma, hence the importance of ordering a biopsy at progression. Many studies are underway to better understand these resistance mechanisms and find treatment strategies to appropriately treat patients, including using new combinations or novel therapeutic agents.

Post-progression on osimertinib, the MARIPOSA-2 trial studied either chemotherapy, amivantamab–chemotherapy, or chemotherapy–amivantamab–lazertinib [13]. PFS was longer with amivantamab–chemotherapy compared to chemotherapy alone (6.3 vs. 4.2 months, HR 0.48). Despite having a better PFS (8.3 months) with the combination of the three drugs, hematologic adverse events were more common, so this option is not considered appropriate. The survival data are still not mature.

After progression on osimertinib and platinum-based chemotherapy, the PALOMA-3 study demonstrated that subcutaneous amivantamab–lazertinib was noninferior to intravenous amivantamab–lazertinib [14]. With subcutaneous amivantamab, a better safety profile was seen with reduced infusion-related reactions and venous thromboembolism, with a shorter median administration time.

Other options still under study after TKI and platinum doublet chemotherapy include antibody–drug conjugates (ADC): datopotamab deruxtecan or patritumab deruxtecan or a combination with another TKI if MET amplification is positive, including osimertinib–savolitinib or osimertinib–tepotinib. Recently, datopotamab deruxtecan (Dato-DXd) has become available for patients harboring EGFR mutations. The FDA approved it as a breakthrough designation based on the pooled analyses of Tropion-Lung01 and 05 data, where patients with AGA received Dato-DXd [15]. EGFR patients seemed to derive the most benefit compared to other patients, with favorable PFS and OS trends.

Immunotherapy for EGFR mutations is not recommended. This was more formally shown in the KEYNOTE-789 where pembrolizumab was added to chemotherapy after osimertinib did not improve PFS or OS [16].

### 3.2. Adjuvant Therapy

Post-thoracic surgery, patients with EGFR mutations usually receive adjuvant platinum-based chemotherapy for four cycles, especially if the tumor size exceeds 4 cm or if positive lymph nodes N1 and/or N2 were found during surgery. There is interest in targeting the EGFR mutation post-operatively. The use of gefinitib (CTONG trial) did not improve outcomes following standard adjuvant chemotherapy [17]. The ADAURA trial studied the addition of osimertinib [18].

After chemotherapy, which was not mandatory, the ADAURA trial studied patients who were randomized to osimertinib or placebo for 3 years for tumors measuring more than 3 cm or lymph nodes positive for N1 or N2. Disease-free survival (DFS) at 24 months was 89% in the osimertinib versus 52% in the placebo group (HR 0.20). In 2023, the 5-year overall survival results were published [19], again demonstrating a significant benefit, 88% versus 78% (HR 0.49). Adjuvant osimertinib is now a standard of care after adjuvant chemotherapy, if applicable. It remains to be seen if 3 years of osimertinib is the optimal duration for TKI treatment. Studies are underway to better determine if a longer time on osimertinib can improve outcomes.

### 3.3. Post-Chemoradiotherapy for Stage III

A standard of care after definitive chemoradiotherapy (CRT) for unresectable stage III non-small cell lung cancer is immunotherapy with durvalumab according to the PACIFIC trial [20]. Unfortunately, EGFR responds less to durvalumab than other patients. The addition of durvalumab may also be deleterious and is not recommended. As for post-operative osimertinib, the addition of osimertinib post-CRT may add value. The LAURA trial assigned EGFR patients without progression post-CRT to either osimertinib or placebo until disease progression [21]. Osimertinib significantly improved median PFS (39.1 vs. 5.6 months, HR 0.16). Overall survival is not yet mature. It will become a standard treatment after CRT, when available.

## 4. EGFR Uncommon Mutations

EGFR uncommon mutations typically refer to variations such as G719X, S768I, and L861Q. Treatment options are limited, but second/third-generation TKIs can be used. The most widely used TKI is afatinib, as it has shown the most benefit in this rare patient subgroup [22]. Some data also show that osimertinib may add some benefit to the patients and could be a good alternative [23,24]. The UNICORN trial showed that, while PFS was shorter for some of the uncommon mutations [25], it was still better than chemotherapy and potentially better than other TKIs. That said, osimertinib is not indicated in this patient population, and its use remains on a case-by-case basis.

## 5. EGFR Mutations: Exon 20 Insertions

EGFR exon 20 insertions are also more common in women, never smokers, Asians, and patients with an adenocarcinoma. However, EGFR exon 20 insertions do not respond to osimertinib or any previous EGFR TKIs already mentioned for common EGFR mutations. In the CHRYSALIS phase I study, amivantamab as a second line or more had a median PFS of 8.3 months after progression on platinum-based chemotherapy [26]. Even if the data is not compared to standard care or best supportive care, patients seem to derive some benefit from amivantamab in this setting.

In the PAPILLON trial, amivantamab combined with chemotherapy in the first line had a better outcome than chemotherapy alone [27]. PFS was significantly longer in the amivantamab–chemotherapy group (11.4 vs. 6.7 months, HR 0.40). Interim OS analysis is not statistically significant (HR 0.67). The combination of chemotherapy–amivantamab will most likely become standard therapy, as few therapeutic options are available for these patients, who have a poor prognosis.

Other treatments under study are TKI, zipalertinib, sunvozertinib, and furmonertinib. Unfortunately, poziotinib and mobocertinib were not promising enough to continue their development.

## 6. ALK Fusions

As for EGFR mutations, ALK fusions are more common for never smokers and patients with adenocarcinoma histology. They are associated with an increase in venous thromboembolism (odds ratio 2.10) according to a recent meta-analysis [28].

### 6.1. Metastatic Disease

In 2013, the results of the trial PROFILE 1007 with an oral ALK TKI crizotinib were published for advanced ALK-positive lung cancer, comparing crizotinib to the usual standard-of-care chemotherapy [29]. Patients had received one prior platinum-based regimen. The median PFS was 7.7 months versus 3.0 months (HR 0.49) in favor of crizotinib. In PROFILE-14, crizotinib was superior to platinum-based chemotherapy [30]. Similarly, in the ASCEND-4 trial, ceritinib was better than platinum-based chemotherapy [31]. Since then, several other treatments have been developed (Table 3).

In 2017, alectinib was compared to crizotinib in the ALEX trial [32]. This second-generation TKI had a better central nervous system (CNS) efficacy than crizotinib. PFS was improved at 12 months, at 68.4% with alectinib versus 48.7% with crizotinib (HR 0.47). Only 12% of patients in the alectinib group had a CNS progression, compared to 45% in the crizotinib group (HR 0.16). Adverse events were also lower in the alectinib group. The long-term data showed an impressive PFS of 34.8 months with alectinib versus 10.9 months with crizotinib (HR 0.43) [33].

Brigatinib was also compared to crizotinib in the ALTA-1L trial [34]. Brigatinib at 12 months had a better PFS, at 67% versus 43% (HR 0.49). This drug also crosses the blood–brain barrier. The final results revealed that the median PFS was 24.0 months versus 11.1 months (HR 0.48) [35].

Similarly, lorlatinib was compared to crizotinib in the CROWN trial [36]. At 12 months, PFS was 78% with lorlatinib and 39% with crizotinib (HR 0.28). Adverse events, particularly neurological and altered lipid levels, were more common with lorlatinib. Five-year outcomes were published in 2024, showing that the 5-year PFS was 60% with lorlatinib and 8% with crizotinib [37]. This is the longest PFS ever reported with any single-agent molecular targeted treatment in advanced non-small cell lung cancer and other metastatic solid tumors. There is also prolonged intracranial efficacy, with a median time to intracranial progression not reached with lorlatinib and 16.4 months with crizotinib (HR 0.06).

No prospective trial compared alectinib, brigatinib, or lorlatinib. Meta-analyses have shown that alectinib is probably the safest drug [38,39]. Lorlatinib had the best efficacy regarding PFS for global patients, followed closely by alectinib and brigatinib [39]. However, long-term data with lorlatinib favor this drug as a first choice, considering that adverse effects must be particularly monitored. The toxicities most commonly described with lorlatinib are metabolic (hypercholesterolemia/hypertriglyceridemia), gastrointestinal (diarrhea), hepatotoxicity, peripheral edema, and neurocognitive disturbances. Dose reduction in the first 4 months did not impact the efficacy of lorlatinib, and it is suggested to dose-reduce to allow for better toxicity management, hence also continuing the TKI. Another promising molecule is NVL-655, according to the phase 1 ALKOVE-1 study presented at the 2024 European Society of Medical Oncology Congress [40].

### 6.2. Adjuvant Therapy

In the ALINA trial, alectinib was compared to chemotherapy after surgery for ALK-positive cancer [41]. The tumors included were 4 cm or more or those that had a positive lymph node in N1 and/or N2. The disease-free survival at 2 years was 93.6% for alectinib versus 63.7% for chemotherapy (HR 0.24). Alectinib was also associated with a decrease in CNS metastases. Grade 3 or 5 adverse events were less common with alectinib than chemotherapy (18.0% versus 27.5%). Some experts may be uncomfortable with withholding chemotherapy for these patients. It remains to be seen with the long-term follow-up if chemotherapy is necessary in this patient population.

## 7. ROS1 Fusions

As for EGFR mutations and ALK fusions, ROS1 is more common for non-smokers and patients with an adenocarcinoma histology. ROS1 patients have an increase in venous (odds ratio 3.15) [28].

Crizotinib, in 2014 in a phase 1 study, PROFILE 001, showed that the response rate was 72% and the median PFS 19.3 months [42]. A subsequent follow-up had a median OS of 51.4 months [43]. Unfortunately, this drug is less effective for brain metastasis, with little central nervous system penetration. Entrectinib became a new standard of care in 2020 with the publication of an integrated analysis of three phase 1-2 trials (ALKA-372-001, STARTRK-1, and STARTRK-2) [44]. Entrectinib has a well-documented blood–brain barrier penetration. The median duration of response was 24.6 months. Long-term efficacy was also significant for entrectinib, with a median PFS of 15.7 months and a median OS of 47.8 months [45]. In patients with measurable CNS metastases, the intracranial overall response (ORR) rate was 80%, and the intracranial PFS was 8.8 months. A phase 3 trial is underway to compare entrectinib and crizotinib in the first line. The results should be available in 2027.

Other promising drugs are lorlatinib and repotrectinib. Repotrectinib was evaluated in the TRIDENT-1 phase 1-2 trial; it is a next-generation ROS TKI with activity against resistance mutations such as ROS G2032R [46]. The median PFS for repotrectinib was 35.7 months. The preliminary data also look favorable for zidesamtinib (NVL-520), as evaluated in the ARROS-1 trial [47].

## 8. BRAF V600E Mutations

BRAF mutations can arise in different populations, with no specific phenotype. It can be detected in older patients, with or without a smoking history, and is slightly more frequent in women. BRAF V600E is the most prevalent, occurring in 50% of BRAF mutations.

In 2017, a phase 2 trial demonstrated that the combination of dabrafenib plus trametinib in patients with previously untreated metastatic BRAF V600E mutations achieved an overall response of 64% [48]. The updated 5-year survival rate of dabrafenib plus trametinib was 22%, with a median OS of 17.3 months and a median PFS of 10.8 months [49]. The overall response rate was 63.9%. Similar results were reported for previously treated patients with platinum-based chemotherapy. Dabrafenib is a BRAF inhibitor, and trametinib is a MEK inhibitor. The main toxicities with BRAF/MEK inhibition are pyrexia, gastrointestinal (nausea, diarrhea), cutaneous (rash), and peripheral edema. Access to BRAF inhibitors is variable depending on the patient’s residence country/region.

In a subsequent phase 2 trial, the PHAROS study, the combination of encorafenib and binimetinib had an overall response rate of 75%, and the median PFS was not evaluable [50]. Encorafenib is a BRAF inhibitor, and binimetinib is a MEK inhibitor. At the progression of TKIs, chemotherapy with immunotherapy is the recommended option.

## 9. MET Exon 14-Skipping Mutations

MET exon 14-skipping mutations (METex14) also do not have a specific phenotype. It can arise in older patients, with or without a smoking history, and is slightly more frequent in women. It should be noted that it can occur in 20–30% of sarcomatoid tumor subtypes.

METex14 should be differentiated from MET amplification. The mechanisms of action are different. MET-skipping mutations involve exon 14 of the MET gene being skipped during transcription, which leads to the loss of a critical tyrosine kinase domain, resulting in dysregulated MET activation. MET amplification refers to an increase in the number of copies of the MET gene, which leads to overexpression of the MET receptor tyrosine kinase. This causes activation of downstream pathways (including PI3K and RAS). MET amplification is a common resistance mechanism in EGFR-mutated patients. It can also be found in EGFR wild-type lung cancer. These findings are associated with a poor prognosis.

We will first discuss METex14 inhibitors. Two trials led to approvals for capmatinib and tepotinib, which are selective METex14 inhibitors. Another option is crizotinib, where the PROFILE 1001 trial showed a median PFS of 8 months [51], but it is not approved in many countries for this indication. As it has little CNS penetration, it does not make it a good therapeutic choice.

In the phase 2 trial GEOMETRY Mono-1, capmatinib showed a significant benefit, with treatment-naive patients having an overall response rate (ORR) of 68.3%, a median PFS of 12.4 months, and a median OS of 25.49 months [52]. In the larger phase 2 trial VISION, treatment-naive patients treated with tepotinib had an ORR of 57.3% with a median duration response of 46.4 months [53,54]. In the never-treated patients with METex14 detected on a tissue biopsy, the median PFS with tepotinib was 15.9 months, and the median OS was 29.7 months. CNS activity seems to be described as well for tepotinib. For capmatinib and tepotinib, the median PFS/OS was higher in the treatment-naive patients compared to the patients who have received the drugs in the second line or more.

Both trials are single-arm, non-comparative trials. Indirect comparisons show that targeted therapy outperforms the standard of care (chemotherapy with or without immunotherapy) in this patient population. The main toxicities with MET inhibitors are peripheral edema, gastrointestinal (nausea, diarrhea), and hepatotoxicity, which are all manageable. For peripheral edema, a proactive approach is recommended with symptom monitoring, lifestyle modifications, and compressive stockings. If it worsens and impacts functioning, pausing the medication and dose reduction is needed.

The promising future treatment in the second line for METex14 is ensartinib, which is showing encouraging anti-tumor activity and a manageable safety profile [55].

For MET amplification, no treatment is currently approved. As previously mentioned, MET amplification is a common resistance mechanism after EGFR TKIs. The combination of savolitinib and osimertinib is promising according to the phase 2 study SAVANNAH, with an ORR of 49%, a median duration of response of 9.3 months, and a median PFS of 7.1 months [56]. A phase 3 trial called SAFFRON is underway with savolitinib in combination with osimertinib versus platinum-based doublet chemotherapy in participants with EGFR-mutated, MET-overexpressed, and/or amplified NSCLC with prior progression on osimertinib.

Similarly, in a phase 2 trial named INSIGHT 2, tepotinib and osimertinib were combined in patients with EGFR-mutation NSCLC with MET amplification following progression on first-line osimertinib. The confirmed ORR was 50% [57].

## 10. RET Fusions

RET fusions occur more often in non-smokers with adenocarcinoma. Two trials led to approval for RET rearrangement patients in the first line, ARROW, and LIBRETTO.

The phase 1/2 study ARROW with pralsetinib had a response rate of 61% in patients treated with previous platinum-based chemotherapy and 70% in treatment-naïve patients [58]. A recent update showed that the median PFS with pralsetinib was 16.5 months for patients with previous treatment and 13.0 months for treatment-naive ones [59]. A phase 3 AcceleRET-Lung study is currently active comparing pralsetinib with platinum-based chemotherapy with or without pembrolizumab, but recruitment is completed.

A phase 1-2 LIBRETTO-001 trial with selpercatinib had an ORR of 85% in the first line and 64% in the second line or more [60]. The median intracranial PFS was 13.7 months at a median duration of follow-up of 11.0 months. The final data showed for treatment-naïve patients a median PFS of 22.0 months, and with a median follow-up of 37.1 months, the median OS was not reached [61,62]. The phase 3 LIBRETTO-431, comparing selpercatinib vs. platinum-based chemotherapy with or without pembrolizumab in the first line, confirmed the superiority of targeted compared to the usual treatment [63]. The median PFS was 24.8 months with selpercatinib and 11.2 months with a control treatment. Selpercatinib also demonstrated improved outcomes in CNS disease. This trial highlights the importance of starting with targeted therapy first whenever possible, with the option of treatment with chemotherapy with/without immunotherapy at progression. The main toxicities for RET inhibitors are hepatotoxicity, gastrointestinal (nausea, mucositis, and diarrhea), hypertension, proteinuria, and QT interval prolongation, which requires ECG monitoring. Interstitial lung disease (ILD) is a rare but severe adverse event.

The phase 3 trial LIBRETTO-432, comparing selpercatinib versus placebo as an adjuvant treatment, is currently underway. Patients should have priorly received the usual adjuvant treatment with chemotherapy/immunotherapy.

## 11. KRAS G12C Mutations

KRAS G12C mutations are still treated with the current standard of care of immunotherapy with or without chemotherapy in the first line. They are more frequently discovered in smokers compared to other actionable genomic alterations. Other KRAS G12 mutations are discovered by NGS testing, notably G12V, G12A, and G12D, but are not actionable for the moment. After immunotherapy and platinum-based chemotherapy, two drugs have demonstrated a benefit: sotorasib and adagrasib.

The phase 2 trial CodeBreaK100 led to the approval of sotorasib with an ORR of 37.1%, a median PFS of 6.8 months, and a median OS of 12.5 months [64]. The subsequent phase 3 study CodeBreaK200 compared sotorasib to usual care with docetaxel [65]. The results showed that the ORR was 28.1% with sotorasib and 13.2% with chemotherapy. The median PFS was 5.6 months with the targeted therapy versus 4.5 months with docetaxel (HR 0.66). Overall survival was not significantly different between sotorasib and docetaxel (mOS 10.6 months and 11.3 months, HR 1.01). Sotorasib had fewer adverse events, and quality of life seemed to improve with the use of sotorasib. Real-world evidence also mirrors the findings of the main sotorasib trials, even with patients with a poorer performance status or multiple lines of treatment administered.

The phase 1-2 KRYSTAL-1 study with adagrasib revealed an ORR of 42.9%, a median PFS of 6.5 months, and a median OS of 12.6 months [66]. The intracranial ORR was 33.3%. The phase 3 KRYSTAL-12 trial was presented at ASCO 2024 [67]. The median was PFS 5.49 months with adagrasib versus 3.84 months with docetaxel (HR 0.58). ORR by BICR was also significantly higher with adagrasib compared to docetaxel (31.9% versus 9.2% (odds ratio 4.68). Intracranial ORR was 40% with the targeted therapy compared to 11% with chemotherapy. The overall survival data are still not mature.

They have become a standard treatment and are now routinely used (if available) after immunotherapy and chemotherapy, with docetaxel being administered afterward. The main toxicities with KRAS inhibitors are gastrointestinal (nausea, diarrhea) and hepatotoxicity. This is worse when TKIs are given just after immunotherapy, with a washout period recommended if possible. Interstitial lung disease (ILD) is a rare but severe adverse event to follow.

Many studies are currently underway for KRAS G12C mutations, including in the first line with KRAS inhibitors given in combination with chemotherapy or immunotherapy. Another KRAS G12C to follow closely is divarasib [68], a more potent inhibitor.

## 12. HER2 or ERBB2 Mutations

ERBB2 mutations, also called HER2 mutations, are more common in women, Asians, never smokers, and young patients.

HER2 (or ERBB2) overexpression should be differentiated from the HER2 (ERBB2) mutant. Contrary to mutations diagnosed by usual next-generation sequencing testing (NGS), they are diagnosed by IHC and should be IHC3+. HER2 overexpression has fewer benefits from treatments than HER2 mutant [69].

In 2025, the first-line treatment for ERBB2 mutation is still a combination of immunotherapy and platinum-based chemotherapy. The second-line treatment that is recommended by most guidelines is an antibody–drug conjugate called trastuzumab deruxtecan (T-DXd), if available. In the phase 2 DESTINY-Lung01 trial for patients who are refractory to standard treatments, an overall response rate of 55%, a median PFS of 8.2 months, and an OS of 17.8 months were observed [70]. In the phase 2 DESTINY-Lung 02, 5.4 mg/kg versus 6.4 mg/kg were compared [71]. Overall response rates were similar, respectively, 49% and 56%. A lower dose of 5.4 mg/kg was associated with fewer adverse effects favoring this option, especially drug-related interstitial pneumonitis. A Phase III trial DESTINY-Lung04 comparing trastuzumab deruxtecan with a combination of pembrolizumab–platinum-based chemotherapy in the first line is currently actively enrolling [72].

Another treatment option is trastuzumab emtasine [73], though not formally indicated in lung cancer. A promising future treatment is zongertinib, an oral TKI targeting ERRB2 mutations [74].

## 13. NTRK Fusions

NTRK fusions are rarely reported; they are more common in young patients, never smokers, and patients with an adenocarcinoma histology. If a TRK inhibitor is available in the first line, it should be prescribed. Otherwise, standard treatment with immunotherapy with or without platinum-based chemotherapy according to PD-L1 should be considered.

Several TKIs have been developed to target NTRK fusions and were studied in a small number of patients. Larotrectinib is a selective TKI that has demonstrated high response rates and durable disease control in NTRK fusion-positive cancers, including NSCLC. In a basket trial including 14 patients with lung cancer, there was an overall response rate of 71% with larotrectinib [75]. An updated analysis presented at the World Conference on Lung Cancer 2024 revealed an overall response rate of 66%, a median duration of response of 34 months, a median PFS of 22 months, and a median survival of 39 months for 32 patients treated with larotrectinib. It was also shown to cross the blood–brain barrier and has intracranial activity [76]. In a similar basket trial including 10 patients with lung cancer, there was an overall response rate of 70% with entrectinib, and it also has intracranial activity [77]. Entrectinib is a multi-kinase inhibitor targeting TRK, ROS1, and ALK. Entrectinib, as previously mentioned, is also approved in NSCLC harboring ROS1 gene fusions. The main toxicities described with TRK inhibitors are neurological (dizziness, ataxia, or neuropathies), gastrointestinal (nausea, diarrhea, or constipation), hepatoxicity, hyperuricemia (potentially leading to gout), and QT interval prolongation, which requires ECG monitoring.

Promising second-generation TRK inhibitors are repotrectininb, selitrectinib, and taletrectinib.

## 14. Conclusions

In conclusion, there has been a revolution in the targeted treatment of lung cancer over the last decade. These new therapies have improved outcomes, including increasing survival rates and quality of life for patients. Treatments are mostly available for the metastatic stages, but the future is also promising for earlier stages. It is necessary to emphasize that molecular testing should be conducted reflexively so that the results can benefit all lung cancer patients, and if possible, all stages of lung cancer. It is also important to check what is available in each jurisdiction/country, as drug reimbursement and health systems vary widely from one country to another and within different regions. Many more potential targets are being described in lung cancer, and multiple novel therapeutic agents are currently being studied.

## Figures and Tables

**Table 1:** Prevalence of actionable genomic alterations in advanced non-squamous non-small-cell lung cancers (NSCLC).

Gene	Alteration	Prevalence
EGFR	-Common mutations (del19, L858R) -Uncommon mutations (G719X, L861Q, S768I -Exon 20 insertions	-15% (50–60% in Asian) -10% -2%
ALK	Fusions	5%
ROS1	Fusions	1–2%
BRAFV600E	Mutations	2%
MET	-Exon 14-skipping mutations -Amplifications	-3% -1–5%
RET	Fusions	1–2%
KRASG12C	Mutations	12%
ERBB2 (HER2)	-Mutations -Gene amplifications -HER2 overexpressions (diagnostic by immunohistochemistry)	-2–5% -2–4% -2–4% (IHC3+) 2–38% (IHC2+)
NTRK	Fusions	0.23–3%

**Table 2:** EGFR tyrosine kinase inhibitors according to their generation.

TKI Generation	Name of Drugs
First-generation TKI	gefitinib, erlotinib
Second-generation TKI	afatinib, dacomitinib
Third-generation TKI	osimertinib, lazertinib

**Table 3:** ALK tyrosine kinase inhibitors according to their generation.

TKI Generation	Name of Drugs
First-generation TKI	crizotinib
Second-generation TKI	ceritinib, alectinib, brigatinib, ensartinib
Third-generation TKI	lorlatinib
